# The cost-effectiveness of Antiretroviral Treatment in Khayelitsha, South Africa – a primary data analysis

**DOI:** 10.1186/1478-7547-4-20

**Published:** 2006-12-06

**Authors:** Susan M Cleary, Di McIntyre, Andrew M Boulle

**Affiliations:** 1 Health Economics Unit, School of Public Health & Family Medicine, University of Cape Town, Anzio Road, Observatory, 7925, Cape Town, South Africa; 2 Infectious Disease Epidemiology Unit, School of Public Health & Family Medicine, University of Cape Town, Anzio Road, Observatory, 7925, Cape Town, South Africa

## Abstract

**Background:**

Given the size of the HIV epidemic in South Africa and other developing countries, scaling up antiretroviral treatment (ART) represents one of the key public health challenges of the next decade. Appropriate priority setting and budgeting can be assisted by economic data on the costs and cost-effectiveness of ART. The objectives of this research were therefore to estimate HIV healthcare utilisation, the unit costs of HIV services and the cost per life year (LY) and quality adjusted life year (QALY) gained of HIV treatment interventions from a provider's perspective.

**Methods:**

Data on service utilisation, outcomes and costs were collected in the Western Cape Province of South Africa. Utilisation of a full range of HIV healthcare services was estimated from 1,729 patients in the Khayelitsha cohort (1,146 No-ART patient-years, 2,229 ART patient-years) using a before and after study design. Full economic costs of HIV-related services were calculated and were complemented by appropriate secondary data. ART effects (deaths, therapy discontinuation and switching to second-line) were from the same 1,729 patients followed for a maximum of 4 years on ART. No-ART outcomes were estimated from a local natural history cohort. Health-related quality of life was assessed on a sub-sample of 95 patients. Markov modelling was used to calculate lifetime costs, LYs and QALYs and uncertainty was assessed through probabilistic sensitivity analysis on all utilisation and outcome variables. An alternative scenario was constructed to enhance generalizability.

**Results:**

Discounted lifetime costs for No-ART and ART were US$2,743 and US$9,435 over 2 and 8 QALYs respectively. The incremental cost-effectiveness ratio through the use of ART versus No-ART was US$1,102 (95% CI 1,043-1,210) per QALY and US$984 (95% CI 913-1,078) per life year gained. In an alternative scenario where adjustments were made across cost, outcome and utilisation parameters, costs and outcomes were lower, but the ICER was similar.

**Conclusion:**

Decisions to scale-up ART across sub-Saharan Africa have been made in the absence of incremental lifetime cost and cost-effectiveness data which seriously limits attempts to secure funds at the global level for HIV treatment or to set priorities at the country level. This article presents baseline cost-effectiveness data from one of the longest running public healthcare antiretroviral treatment programmes in Africa that could assist in enhancing efficient resource allocation and equitable access to HIV treatment.

## Background

Antiretroviral treatment has been shown to be effective in reducing morbidity and mortality in patients infected with HIV in developing countries [1]. However, in sub-Saharan Africa where 25.8 million are HIV-infected [2] only 17% of those in need of ART were using treatment by the end of 2005 [3]. Although progress has been made in extending coverage, the United Nations General Assembly target of universal access to antiretroviral treatment (ART) by 2010 for all in need [4] presents a formidable challenge.

Given the scale of treatment envisaged, the paucity of data estimating the lifetime costs and efficiency of HIV treatment is a serious hindrance to effective planning. In the absence of data, most global estimates of resource needs have been based on normative modelling exercises and in publishing these estimates, authors have urgently recommended primary research into the costs and cost-effectiveness of ART to address these gaps [5,6].

The objectives of this research were to estimate the utilisation and costs of HIV-related healthcare, to estimate lifetime costs, life years and quality adjusted life years (QALYs) and to assess cost-effectiveness from the provider's perspective by comparing treatment and prophylaxis of opportunistic and HIV-related illnesses without antiretrovirals (hereafter "No-ART") to costs and effects when ARVs are used ("ART") based on primary unit cost, utilisation, health-related quality of life (HRQoL) and outcome data from a cohort accessing care in a resource poor setting in South Africa.

## Methods

### Study design

This study undertakes a cost-effectiveness analysis from a provider's perspective. The utilisation of a full range of HIV-related services was calculated using a before and after study design. Full economic costs were calculated using the ingredients and step-down methods. Markov modelling – an approach to extrapolating data [7] – was used to calculate lifetime costs, LYs, QALYs and incremental cost-effectiveness ratios (ICERs). Costs and effects are presented for zero and 3 percent annual discount rates. Multi-way and probabilistic sensitivity analyses were used to assess uncertainty.

### Study population and description of interventions

Patients included in this study live in Khayelitsha, a township on the outskirts of Cape Town characterised by a high proportion of informal housing and lack of access to basic services. The level of unemployment in the area is estimated to be 46% [8]. In April 2000, three HIV clinics were opened within existing public sector clinics to provide treatment and prophylaxis of HIV-related and opportunistic infections and events, counselling and support groups for HIV-positive people. Prophylactic medication included trimethoprim-sulphamethoxazole and fluconazole for eligible patients. Acute infections were managed at the clinics but severely ill patients were referred to secondary and tertiary hospitals. Patients suspected of having tuberculosis (TB) were referred to TB facilities.

In May 2001, the service was extended to include ART for patients with CD4 counts less than 200 cells/μl at any WHO stage or with WHO stage IV and any CD4 level. This was the first public sector programme offering ART in South Africa and experience from this initiative has informed the development of local ART guidelines. ART patients continued to receive treatment and prophylaxis for acute infections and appropriate referrals once initiated on ART.

### Healthcare utilisation

Healthcare utilisation, including HIV clinic visits, TB treatment and inpatient care, was established using a before and after study design. This means that ART patients were used as their own control – the pre-ART period was used to calculate No-ART utilisation while the post-baseline period informed ART estimates. While a clinical trial comparing No-ART to ART would be the gold standard for measuring utilisation, obvious ethical limitations imply that the before and after study design is one of the only possible choices in this context. The delay between the start of the project in 2000 and the introduction of ART in 2001, as well as the limited number of patients on ART in the early years of the project ensure that utilisation prior to initiation of ART is reflective of general HIV care in the absence of ART.

HIV clinic utilisation and associated 95% confidence intervals was calculated from 1,729 patients with 1,146 No-ART patient years and 2,229 ART patient years of follow-up over a median No-ART and ART follow-up of 0.63 years (IQR 0.33–1.32, max 4.35) and 1.03 years (IQR 0.68 – 1.70, max 4.08) respectively. Data on the use of inpatient and tuberculosis care required extensive validation. This validation was undertaken on a sub-sample of 670 patients, with 501 No-ART patient-years and 693 ART patient-years. One would anticipate that patients who died would receive the highest concentration of inpatient care around this time. However, with a before and after study design, by definition there are no deaths in the No-ART group who are in reality a pre-ART group. We have accounted for this by calculating a separate "cost of dying" from a sample of 83 patients who had been using services in the HIV clinics but had died before being able to start ART. These patients were followed up at hospitals to establish their utilisation of inpatient care in the 6-month period preceding death. The same procedure was followed for the 81 patients on ART who died of HIV-related causes. This approach ensures that inpatient utilisation at the time of death is not underestimated.

### Unit costs of clinic visits, inpatient care and tuberculosis treatment

Costing has been undertaken from a provider's perspective. This means that all direct health care costs and costs of non-governmental organisations have been included, but that direct non health care costs (such as patient travel and time costs) have been excluded [9]. Unit costs of health services are defined as the full economic cost per ART or No-ART visit, per inpatient day at tertiary and secondary/district facilities and per tuberculosis case treated. Costing of HIV clinic services was undertaken separately for ART and No-ART visits. Costing of TB treatment was undertaken at the Nyanga clinic, and was supplemented with secondary data [10]. Nyanga clinic was chosen because 67% of TB patients have an HIV-positive diagnosis which is the highest in the area. Khayelitsha patients are referred to Tygerberg Academic Hospital, Groote Schuur Hospital (tertiary) and to GF Jooste Hospital (secondary/district) for inpatient care. Costing of HIV inpatient care was undertaken at Tygerberg and secondary data informed costs for Groote Schuur and GF Jooste [11-13].

Each unit cost has been categorised into patient-specific, clinical staff (medical officers and nurses), overhead and capital components. Patient-specific costs include medicines (curative and prophylactic excluding ARVs which have been calculated separately), laboratory investigations (excluding ART safety and monitoring laboratory tests which have also been calculated separately), imaging and procedures. Mean patient-specific unit costs were calculated by multiplying physical units of resources consumed with their market values (assumed to be equivalent to opportunity costs). The following market values have been used:

• Medicine costs from provincial government tender prices

• Laboratory test costs from National Health Laboratory Services (NHLS). The NHLS is the provider of laboratory services to the public health system.

• Imaging and procedure costs from the Uniform Patient Fee Schedule [14] which details the fees to be charged to private patients in public sector hospitals – private fee scales are based on costs.

Prescriptions for curative and prophylactic medicines and multivitamins were extracted from the records of 60 patients who had been on ART for at least one year. This amounted to a medicine costing sample of 757 visits for No-ART patients and 1,532 visits for ART patients. Tuberculosis patient-specific resource use (medicines, X-rays and diagnostic tests) was based on South African national TB treatment protocols [15]. Additional patient-specific resources that were prescribed to TB patients (acute non-TB medicines) and clinical staff costs for TB initiation and monitoring visits were derived from HIV-positive TB patients. At the tertiary hospital, HIV-related patient-specific resource use was collected by medical officers and entered onto specifically designed data collection tools. Sixty-one patients with 243 inpatient days were included in the analysis. At the secondary/district hospital, patient-specific cost data were derived from a secondary source [11].

We define overhead costs to include recurrent costs that are not directly related to patient numbers including utilities (water, electricity) and non-clinical staff (administrative, cleaning and security personnel). Under the assumption that all patients utilise a similar amount of overheads during each visit or inpatient day, overhead costs have been calculated by establishing overhead expenditure from routine facility accounting data and dividing this by total patient visits and/or inpatient days. All data were measured over an annual period to minimise biases that might result from seasonal variations in expenditure, visits or inpatient days. For hospitals, expenditure was first allocated between inpatient and outpatient departments using the patient-day equivalent method [16] which amounts to assuming that an inpatient day requires just under four-times more overhead resources than an outpatient department or emergency visit. This ratio is based on primary data collection in Western Cape hospitals.

Clinical staff – including medical officers and nurses – are likely to be a key constraint in attaining universal access to HIV-related care [17]. At clinics, clinical staff requirements were estimated by timing 54 ART visits, 94 No-ART visits, and 25 treatment initiation/monitoring visits for HIV-positive patients at the TB clinic. This estimate of the time per clinical consultation was multiplied against the average clinical staff cost per minute (adjusted for annual working days and patient contact hours) to calculate a clinical staff cost per visit. At the inpatient level, it was more difficult to calculate an HIV-specific clinical staff cost owing to restrictions on researcher access to hospital wards. At the secondary hospital, we established the number of full time equivalent doctors and nurses in each relevant ward and their average cost of employment. The resultant cost was split equally between inpatients in these wards. At the tertiary hospital, we established the annual expenditure on medical officers and allocated this to inpatient days using the patient-day equivalent method.

Capital costs relate to the costs of medical equipment, furniture and buildings. Following costing literature [18], these were calculated by establishing the replacement value of each item, estimating the working life and annuatizing using an 8% real interest rate (the return on South African long-term government bonds). The resultant annual cost was allocated to inpatient days and clinic visits using the same method as for overheads.

Costs were expressed in 2003 prices and converted to US$ using an average 2003 exchange rate (US$1 = 7.56 Rands) [19]. Any inflation adjustments used the consumer price index excluding mortgage bonds [20].

### The costs of ARVs and monitoring and safety laboratory investigations

The regimen of choice for patients starting ART in Khayelitsha has changed over time. Whereas previously all patients started on a nucleoside reverse transcriptase inhibitor (NRTI) backbone of zidovudine and lamivudine, this has been replaced by stavudine and lamivudine in line with national ART guidelines [21], and so the nationally recommended NRTI backbone was costed. As the national guidelines allow for flexibility in the choice of non-nucleoside reverse transcriptase inhibitors (NNRTIs -nevirapine or efavirenz) to accompany the NRTI backbone, primary data were used to determine the relative use of NNRTIs. The second-line regimen consists of zidovudine, didanosine and lopinavir/ritonavir. Laboratory investigation requirements have been based on national guidelines [21].

Public sector ARV costs (including delivery costs to the provincial depots) were sourced from the South African national ARV tender [22]. It is expected that this current tender will be extended until end of February 2008 after which time a new tender will be introduced which might alter current prices depending on the outcomes of negotiations between the government and pharmaceutical companies (Liezl Channing, Pharmacist – HIV/AIDS/STI/TB Programmes – Western Cape Provincial Government, personal communication). Laboratory investigation costs have been sourced from the National Health Laboratory Services.

### Study models

Lifetime results are an important consideration in planning the resource needs for scaling-up ART – if life-expectancy is underestimated, this will underestimate the numbers of patients remaining in care which will ultimately lead to severely constrained budgets. Lifetime costs and outcomes have been calculated with the aid of Markov modelling. Markov models consist of mutually exclusive and collectively exhaustive health (Markov) states with transition probabilities describing all possible or relevant movements between these states. Transitions occur after discrete time periods, known as Markov cycles. Markov states are defined such that "patients" in the state have a similar risk of events of importance to the disease in question (e.g. death) and similar costs of healthcare. When the model is constructed, healthcare costs and health outcomes are attached to each Markov state. For example, if one is modelling QALYs using a three-month Markov cycle, 0.25 (representing three months in a year) would be multiplied by the appropriate HRQoL value and attached to each state (with the exception of "dead"). Similarly, the cost of being in each Markov state is computed for the length of the Markov cycle and is attached to the state. When the model is run over a large number of cycles, lifetime costs and outcomes are calculated [7,23].

For the ART model, separate Markov states have been specified for CD4 50–199 cells/μl, and CD4 < 50 cells/μl because these categories have been shown to be associated with different mortality rates in large cohort analyses [24,25]. States have also been stratified according to the amount of time a patient has been on ART. This stratification was required because mortality is concentrated during the first six months on ART and decreases dramatically thereafter [1]. Similarly, the costs of healthcare were found to be higher closer to the time of ART-initiation because patients had higher rates of HIV clinic visits, inpatient care and TB treatment.

To adequately capture the trend in costs and outcomes related to duration on ART, the CD4-based Markov states were sub-divided into temporary states, known as tunnel states. During the first 6 month period, tunnel states were created both for each Markov cycle (i.e. 3-month period) and for each CD4 category. After 6 months on ART, differences related to baseline CD4 levels were less significant, and the CD4 states were merged. However, differences in mortality rates and costs were still significantly related to duration on ART, necessitating the ongoing use of tunnel states for duration periods of between 6 and 48 months on treatment. Separate states for the second-line regimen were also created to capture differences in ARV regimen costs.

The No-ART model was stratified into CD4-based Markov states of CD4 50–199 cells/μl and CD4 < 50 cells/μl, with death as the absorbing state.

**Figure 1:**
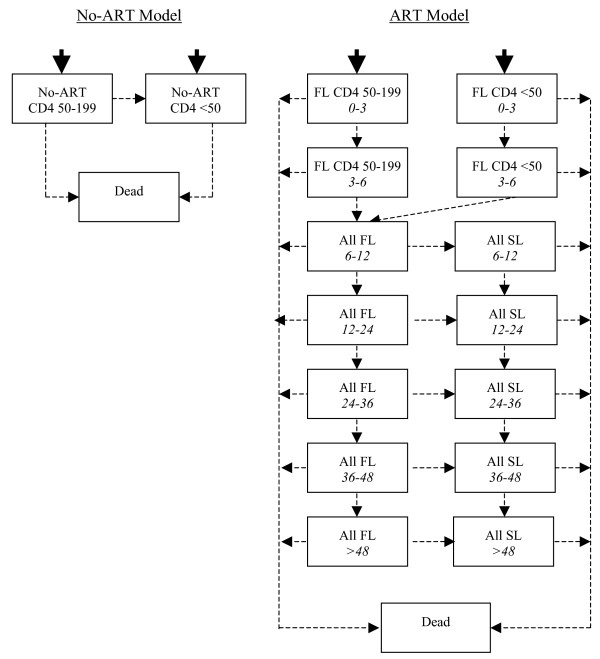
**Markov models for No-ART and ART**. All: All patients. FL: First-line ART regimen; SL: Second-line ART regimen; *0–3*; *3–6*; *6–12*; *12–24; 24–36; 36–48; *and *>48 *refer to months since the initiation of ART

The proportion of patients entering the models with CD4 50–199 cells/μl versus CD4 < 50 cells/μl has been based on the baseline CD4 counts of ART patients in the HIV clinics. Models have been developed in TreeAge Pro^® ^2005 and are depicted in figure 1.

### Transition probabilities

Transition probabilities in a Markov model are required to specify all relevant movements between Markov states – as depicted by the arrows in figure 1. In the ART model, transition probabilities together with 95% confidence intervals were estimated from Kaplan Meier product limit estimates of survival for 1,729 patients accessing ART in the first 48 months of the Khayelitsha programme. A probability of dying was calculated directly from primary data in months 0–3, 3–6, 6–12, 12–24, 24–36 and 36–48. This allowed an accurate specification of the decline in mortality over four years on ART. Patients who were lost to follow-up were treated statistically as deaths. This overestimate of mortality might be more reflective of routine care settings where adherence to treatment might be lower and loss to follow-up might be higher. Probabilities of switching to the second-line regimen were calculated from primary data separately for months 6–12, 12–24, 24–36 and 36–48. No patients switched to second-line between months 0 and 6.

Given that the majority of patients still remain in care at 48 months on ART, the long-term effectiveness of ART, rates of failure of first-line regimens and costs and outcomes after treatment failure will be unknown for the foreseeable future. We have extrapolated death transition probabilities by calculating the average probability of dying between baseline and 48 months. This probability has been applied uniformly to all periods on treatment thereafter. This approach could underestimate life-expectancy which we consider pragmatic given the many uncertainties. Similarly, the second-line transition probabilities were extrapolated by calculating an average probability between baseline and 48 months.

Transition probabilities for the No-ART model were derived from the Cape Town AIDS Cohort, a local natural history cohort of 981 ART-naïve patients who presented at an HIV clinic established at the New Somerset secondary hospital in Cape Town between 1992 and 2000 [26,27]. These secondary data sources provide Kaplan Meier survival estimates for patients with CD4 < 200 cells/μl and CD4 < 50 cells/μl, but not for patients with CD4 50–199 cells/μl. However, data indicates that a CD4 < 50 cells/μl is associated with a hazard ratio for death of 2.28 (p = 0.021) [28]. We used this hazard ratio to estimate the probability of dying with CD4 50–199 cells/μl as equal to the probability of dying with CD4 < 200 cells/μl divided by the hazard ratio. The probability of switching between CD4 50–199 cells/μl and CD4 < 50 cells/μl was estimated to ensure that overall survival was in line with survival with CD4 < 200 cells/μl.

The final death transition probabilities required in each intervention are the probabilities of dying from non-HIV related causes. These were calculated from South African life tables [29] according to the age, gender and socio-economic status of patients included in this analysis.

### Health-related quality of life

This analysis has expressed outcomes as both LYs and QALYs. QALYs are calculated by weighting life-expectancy by a factor relating to the health-related quality of that life (HRQoL) in relation to a number of aspects of health such as functional, physical and emotional status. The overall rationale for using QALYs is that HIV-positive people on ART have been shown to fare more favourably in relation to quality of life as well as life expectancy than those who are not receiving ART (see [30,31]). HRQoL has been measured in a related study [32] using a widely validated instrument called the EQ-5D on a sub-sample of ART patients in the same cohort at baseline 3, 6 and 12 months (n = 95, 97, 98 and 83 respectively). HRQoL data were converted to utilities using health state values derived from a United Kingdom general population survey using the time trade-off method [33].

### Assessing uncertainty

This analysis is subject to uncertainty relating to the data requirements of the study, generalizability of results, extrapolation of data and choice of analytic methods. Uncertainty relating to data requirements has been assessed using probabilistic sensitivity analysis (PSA). This technique propagates parameter and underlying modelling uncertainty through the model by means of first and second-order Monte Carlo simulation. First-order simulations capture the different paths taken by "patients" in the model in order to capture variability associated with the structure of the model. Second-order Monte Carlo simulation captures parameter uncertainty – distributions are specified on all transition probabilities and utilisation variables and a different value from each distribution is chosen during each simulation. When a number of simulations are run, overall parameter uncertainty is captured as confidence intervals around lifetime costs, outcomes and ICERs [34,35]. Simulations have been run using 1,000 distribution values, and each distribution value has been subjected to 10,000 first-order simulations. Distributions for transition probabilities and utilisation rates were specified as triangular distributions spanning the 95% confidence interval for each estimate, with the mode equal to the estimate in each instance.

Uncertainty relating to generalizability of results is concerned with the extent to which the results of this study can be applicable to other settings. Generalizability has been assessed by comparing ART outcomes to other published low income cohorts [1,36] and No-ART outcomes to a review of natural history data [37]. Inpatient and visit utilisation has been compared to a published South African cost-effectiveness analysis [38] and to national guidelines for follow-up of patients on ART [21]. Unit costs have been compared to other South African studies [10,13,39]. Where disagreement between this study and published data has been found, adjustments have been made through the construction of an alternative scenario.

While no extrapolation is required to estimate outcomes for No-ART patients, uncertainty relating to extrapolation is an important problem in the evaluation of ART. This form of uncertainty has been assessed by comparing ART outcomes from this study to other published studies.

The final source of uncertainty relates to analytic methods. These include methods for valuing and measuring HRQoL and methods for discounting costs and outcomes. This study has considered uncertainty relating to HRQoL measurement and valuation by estimating outcomes as LYs and QALYs and by presenting results discounted at zero and 3 percent annual rates.

## Results

### Healthcare utilisation, unit costs and costs in Markov states

During the follow-up period, No-ART patients had 12,508 visits to the HIV clinics, 1,342 inpatient days and 159 treated episodes of tuberculosis. ART patients had 39,450 HIV clinic visits, 840 days in hospital and 86 TB episodes. Patients who died spent between 3 and 6 days in hospital in the six-months leading up to death.

This data (including 95% confidence intervals) is expressed per patient-quarter in each Markov state in table 1. In the ART intervention, health service utilisation was highest for patients with CD4 < 50 cells/μl during the initial stages on treatment, with 10.5 clinic visits including pre-ART work-up, 1.1 days in hospital and 0.08 tuberculosis cases in the first 3 months. Patients commencing ART with CD4 50–199 cells/μl had 9.7 clinic visits, 0.6 days in hospital and 0.06 cases of tuberculosis. All healthcare utilisation dropped significantly as duration on ART increased with only 2.6 clinic visits, 0.1 inpatient days and 0.02 tuberculosis cases per patient-quarter in the third year. Sixty-one percent of patients received a first-line regimen of stavudine, lamivudine and efavirenz and the remainder received stavudine, lamivudine and nevirapine. All patients received zidovudine, didanosine and lopinavir/ritonavir in the second-line regimen.

**Table 1:** Quarterly healthcare utilisation and total costs in each health state including antiretrovirals and associated laboratory investigations (US$)

Health state	Clinic visits		Inpatient days		Tuberculosis treatment		ARV Cost (95% CI)	Safety and monitoring laboratory costs	**Cost per Markov state ** **(95%CI)**	Additional cost for dying patients	
					
	*Mean visits* *(95%CI)*	Cost (95%CI)	*Mean IP days* *(95%CI)*	Cost (95%CI)	*Mean cases* *(95%CI)*	Cost (95%CI)				*Mean IP days* *(95%CI)*	Cost (95%CI)
ART CD4 < 50 cells/μl months 0–3	*10.5* *(10.2–10.7)*	199 (194–204)	*1.09* *(0.96–1.25)*	177 (155–201)	*0.08* *(0.05–0.12)*	48 (30–76)	72.7 (70.2–75.1)	52	**548** **(502–609)**	*4.0* *(1.7–6.2)*	704 (306–1102)

ART CD4 < 50 cells/μl months 3–6	*3.7* *(3.5–3.8)*	70 (67–73)	*0.77* *(0.66–0.90)*	125 (107–146)	*0.04* *(0.02–0.08)*	25 (13–49)	72.7 (70.2–75.1)	0	**293** **(257–343)**	*4.0* *(1.7–6.2)*	704 (306–1102)

ART CD4 50–199 cells/μl months 0–3	*9.7* *(9.5–9.9)*	184 (181–188)	*0.6* *(0.52–0.7)*	98 (84–114)	*0.06* *(0.04–0.1)*	38 (24–60)	72.7 (70.2–75.1)	52	**444** **(410–488)**	*4.0* *(1.7–6.2)*	704 (306–1102)

ART CD4 50–199 cells/μl months 3–6	*3.5* *(3.3–3.6)*	66 (64–68)	*0.12* *(0.09–0.17)*	20 (14–27)	*0.02* *(0.01–0.04)*	11 (4–26)	72.7 (70.2–75.1)	0	**169** **(152–196)**	*4.0* *(1.7–6.2)*	704 (306–1102)

First-line ART months 6–12	*3.6* *(3.5–3.7)*	69 (67–70)	*0.21* *(0.18–0.24)*	33 (29–39)	*0.02* *(0.01–0.03)*	13 (8–21)	72.7 (70.2–75.1)	24	**212** **(198–229)**	*4.0* *(1.7–6.2)*	704 (306–1102)

Second-line ART months 6–12	*3.6* *(3.5–3.7)*	69 (67–70)	*0.21* *(0.18–0.24)*	33 (29–39)	*0.02* *(0.01–0.03)*	13 (8–21)	238	28	**381** **(370–397)**	*4.0* *(1.7–6.2)*	704 (306–1102)

First-line ART months 12–24	*2.7* *(2.65–2.8)*	52 (50–53)	*0.1* *(0.08–0.13)*	17 (14–20)	*0.02* *(0.01–0.03)*	12 (8–20)	72.7 (70.2–75.1)	24	**178** **(167–193)**	*4.0* *(1.7–6.2)*	704 (306–1102)

Second-line ART months 12–24	*2.7* *(2.65–2.8)*	52 (50–53)	*0.1* *(0.08–0.13)*	17 (14–20)	*0.02* *(0.01–0.03)*	12 (8–20)	238	28	**347** **(338–360)**	*4.0* *(1.7–6.2)*	704 (306–1102)

First-line ART months 24–36	*2.6* *(2.5–2.7)*	49 (47–51)	*0.1* *(0.08–0.13)*	17 (14–20)	*0.02* *(0.01–0.03)*	12 (8–20)	72.7 (70.2–75.1)	24	**175** **(164–191)**	*4.0* *(1.7–6.2)*	704 (306–1102)

Second-line ART months 24–36	*2.6* *(2.5–2.7)*	49 (47–51)	*0.1* *(0.08–0.13)*	17 (14–20)	*0.02* *(0.01–0.03)*	12 (8–20)	238	28	**344** **(335–358)**	*4.0* *(1.7–6.2)*	704 (306–1102)

First-line ART beyond 36 months	*2.8* *(2.6–3.0)*	52 (49–56)	*0.1* *(0.08–0.13)*	17 (14–20)	*0.02* *(0.01–0.03)*	12 (8–20)	72.7 (70.2–75.1)	24	**179** **(165–196)**	*4.0* *(1.7–6.2)*	704 (306–1102)

Second-line ART beyond 36 months	*2.8* *(2.6–3.0)*	52 (49–56)	*0.1* *(0.08–0.13)*	17 (14–20)	*0.02* *(0.01–0.03)*	12 (8–20)	238	28	**348** **(337–363)**	*4.0* *(1.7–6.2)*	704 (306–1102)

No-ART CD4 < 50 cells/μl	*3.4* *(3.2–3.5)*	66 (63–68)	*0.7* *(0.64–0.75)*	113 (104–122)	*0.1* *(0.07–0.13)*	60 (46–79)	N/A	N/A	**239** **(213–269)**	*5.7* *(3.5–8.0)*	1023 (626–1422)

No-ART CD4 50–199 cells/μl	*2.6* *(2.5–2.6)*	51 (49–52)	*0.3* *(0.28–0.32)*	49 (45–52)	*0.07* *(0.06–0.09)*	45 (37–55)	N/A	N/A	**145** **(132–159)**	*5.7* *(3.5–8.0)*	1023 (626–1422)

IP: Inpatient days

No-ART patients with CD4 < 50 cells/μl had 3.4 clinic visits, 0.7 inpatient days, and 0.1 tuberculosis cases per patient-quarter. Patients with CD4 50–199 cells/μl used less health care. All patients spent between 4 and 6 days in hospital prior to death.

Full economic unit costs for HIV clinic visits, inpatient care and TB treatment are presented in table 2. The cost per HIV clinic visit was US$19 for patients on ART and US$20 for those not on ART. The average cost per inpatient day was US$267 at the tertiary hospital and US$119 at the district/secondary hospital. Seventy-one percent of referrals were to the latter, leading to an average weighted cost of US$162 per inpatient day. The cost per tuberculosis case treated was US$622. The proportion of each unit cost relating to doctors and nurses ranged between 4% for TB treatment and 34% for ART clinic visits. The annual cost of the efavirenz containing first-line regimen was US$438 per annum whereas the nevirapine containing regimen cost US$162 per annum. Second-line was more than double the cost of first-line, at US$952 per annum. Laboratory investigations including monitoring (CD4 and viral load) and safety tests (ALT, full blood counts, fasting cholesterol and triglyceride and fasting glucose as required for each ARV) cost around US$25 per patient-quarter (see table 1).

**Table 2:** Unit costs of clinic visits, inpatient days and tuberculosis treatment (US$)

**Cost categories**	**Clinic visits**		**Inpatient care**		**TB treatment**
		
	**ART**	**No-ART**	**Tertiary**	**Secondary**	
Patient-specific	1.65	5.57	28.97	27.66	100.80
Clinical staff	6.64	6.12	35.93	17.86	31.07
Overheads	9.41	5.80	125.46	54.05	488.10
Capital	1.63	1.44	102.91	31.44	1.57
**Unit cost**	19.33	18.92	293.27	131.02	621.54

The cost per patient-quarter in each Markov state has been calculated by multiplying healthcare utilisation against unit costs (table 1). ART costs were highest for patients with CD4 < 50 cells/μl during the first three months on treatment, at US$548 excluding costs for patients who died. Costs remained steady at just over US$170 per quarter from 12 months onwards while patients remained on first-line, but increased to over US$340 per quarter when second-line treatment was initiated. No-ART costs in the CD4 < 50 cells/μl category were US$239 per patient-quarter excluding patients who died. A mean cost of over US$1000 was incurred at hospitals for No-ART patients during the period preceding death.

### Effectiveness

Table 3 provides an overview of transition probabilities. Sixty-three percent of patients initiated ART with CD4 50–199 cells/μl and the remainder with CD4 counts < 50 cells/μl. Similar results have been found in other developing country cohorts [1]. No patients were changed to second-line during the first 6 months, but by 48 months, 16% of those surviving had switched. Deaths were concentrated in the first year. At six months on ART, 83.9% of the 643 patients initiating with a CD4 count < 50 cells/μl were alive in comparison with 93.9% of those initiating with CD4 50–199 cells/μl. The product limit estimate of survival was 86.9%, 83.4% and 76.2% at 12, 24 and 48 months respectively. Fifty percent of No-ART patients with CD4< 200 cells/μl were alive at 24 months. HRQoL values increased from 0.7 at baseline to 0.85 by 12 months on ART as shown in table 4.

**Table 3:** Transition probabilities (per three-month period) and data sources

**Input parameters**	**Data sources**	**Transition probability (range)**
**CD4 category at baseline for ART and No-ART**		
CD4 < 50 cells/ml		0.372 (0.349–0.395)
CD4 50–199 cells/ml		0.628 (0.605–0.651)
**Probabilities of transitioning between alive Markov states**		
*First-line regimen to second-line regimen*		
0–6 months	N/A – no patients switched	
6–12 months	0.48% switched by 12 months	0.002 (0.001–0.006)
12–24 months	4.66% switched by 24 months	0.011 (0.007–0.015)
24–36 months	11.73% switched by 36 months	0.019 (0.014–0.026)
36–48 months	15.9% switched by 48 months	0.012 (0.005–0.024)
>48 months	Average over 0–48 months	0.011 (0.007–0.017)
*No-ART, CD4 50–199 cells/ml to CD4 < 50 cells/ml*		
all quarters	Calculated to ensure 50% surviving at 24 months	0.040 (0.026–0.043)
**Probabilities of transitioning to dead Markov states**		
*ART CD4 < 50 cells/ml*		
0–3 months	86.9% surviving at 3 months	0.131 (0.107–0.159)
3–6 months	83.9% surviving at 6 months	0.034 (0.031–0.038)
*ART CD4 50–199 cells/ml*		
0–3 months	95.9% surviving at 3 months	0.041 (0.030–0.054)
3–6 months	93.9% suriviving at 6 months	0.021 (0.019–0.024)
*All patients on ART, irrespective of regimen*		
6–12 months	86.9% suriviving at 12 months	0.018 (0.017–0.020)
12–24 months	83.4% suriviving at 24 months	0.010 (0.008–0.012)
24–36 months	79.5% surviving at 36 months	0.012 (0.009–0.016)
36–48 months	76.2% suriviving at 48 months	0.010 (0.005–0.017)
>48 months	Average over 0–48 months	0.017 (0.013–0.021)
*No ART CD4 count < 50 cells/ml*		
all quarters	20% surviving at 24 months^1^	0.182 (0.147–0.227)
*No ART CD4 50–199 cells/ml*		
all quarters	50% surviving at 24 months with CD4 < 200 cells/ml^2 ^divided by hazard ratio^3^	0.039 (0.034–0.043)

^1 ^No-ART survival with CD4 < 50 cells/μl from Post, Wood et al [52]

^2 ^No-ART survival with CD4 < 200 cells/μl from Badri, Bekker et al [27]

^3 ^Hazard ratio for death in CD4 < 50 versus 50–199 cells/μl from Coetzee, Hildebrand et al [26]

**Table 4:** HRQoL values

**Health state**	**Value**
ART 0–3 months	0.71
ART 3–6 months	0.81
ART 6–12 months	0.82
ART >12 months	0.85
No-ART	0.71

HRQoL measurements from Jelsma et al [32] and values from Dolan et al [33].

### Lifetime costs, outcomes, cost-effectiveness and probabilistic sensitivity analysis

**Figure 2:**
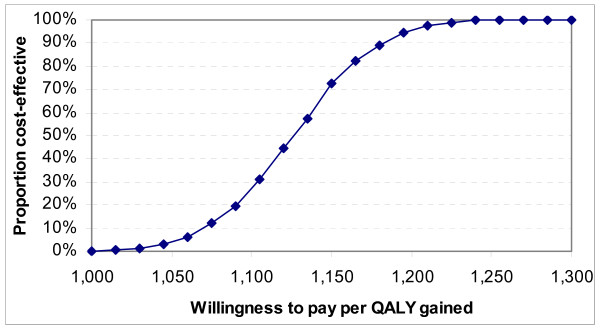
Cost-effectiveness acceptability curve.

Table 5 shows lifetime costs, outcomes and cost-effectiveness results. Undiscounted life years were 2.9 and 12.9 while discounted QALYs (3 percent annual rate) were 1.9 and 8.0 for No-ART and ART respectively. Discounted per-patient lifetime costs were US$2,743 for No-ART versus US$9,435 for ART. The discounted ICER on ART was US$1,102 per QALY gained. The ICER was lower per LY gained, and slightly higher when costs and effects were discounted at a zero rate. Sensitivity around the discounted incremental cost per QALY gained is summarized in a cost-effectiveness acceptability curve in Figure 2. This curve shows the proportion of ICERs derived through probabilistic sensitivity analysis that are lower than alternative levels of willingness to pay per QALY gained. While no simulations produced ICERs below US$1,000, all simulations indicate that ART would be cost-effective at a willingness to pay of US$1,300 per QALY gained.

**Table 5:** Lifetime costs (US$), effectiveness and ICERs of ART compared to No-ART

**Treatment option**	Lifetime costs (95%CI)	Outcomes		ICER^†^	
		
		Life Years (95%CI)	QALYs (95%CI)	Life Years (95%CI)	QALYs (95%CI)
**Undiscounted**					
No-ART	2,966 (2,611–3,343)	2.9 (2.6–3.3)	2.1 (1.8–2.3)		
ART	13,191 (11,167–16,056)	12.9 (11.1–15.2)	10.8 (9.1–12.5)	1,023 (958–1,116)	1,166 (1,092–1,279)
**Discounted**					
No-ART	2,743 (2,414–3,057)	2.7 (2.4–3.0)	1.9 (1.7–2.1)		
ART	9,435 (8,414–10,891)	9.5 (8.5–10.7)	8.0 (7.3–8.6)	984 (913–1,078)	1,102 (1,043–1,210)

Lifetime costs and effectiveness are per patient in each group.

† The ICER is calculated as incremental lifetime costs divided by incremental effectiveness.

The results of probabilistic sensitivity analysis are presented as confidence intervals around these results (see table 5). These indicate that there are distinct increases in lifetime costs and outcomes from implementing ART as opposed to No-ART.

### Results from multi-way sensitivity analysis

We compared our results to those of similar studies to identify which values to adjust in the sensitivity analysis. Given the many uncertainties surrounding the extrapolation of outcomes on ART, a review of studies using Markov modelling was specifically undertaken. In these studies, undiscounted outcomes on ART ranged between 5.8 [40] and 18.8 LYs [38] while the majority of studies calculated outcomes between 14 and 15 LYs [41-43]. In those studies that only presented outcomes that were discounted (all at 3% per annum) the lowest estimate of LYs was 5.3 [44] but most studies reported outcomes of 10–11 years [45,46]. ART outcomes in this study are therefore comparable with those reported in other modelling studies.

In terms of survival on ART, our primary data indicated 86.9%, 83.4% and 76.2% surviving at 12, 24 and 48 months and extrapolated results indicated 71% surviving at 60 months. These outcomes are slightly worse than results from a 7-year Senegalese cohort study [36] which indicated 88.3%, 82.6% and 75.4% at 12, 24 and 60 months respectively, but given the higher baseline CD4 counts of the Senegalese patients, results are comparable. HRQoL values were also highly comparable to estimates from another Cape Town cohort where HRQoL was measured with the SF36 instrument and valued using a United Kingdom-based standard gamble algorithm (see [38]). Unit costs were also similar to other local studies [10,13,39].

Although our results are in line with the literature, most studies in the literature themselves are derived from non-routine settings. We have therefore constructed an alternative scenario to give insights into potential costs and outcomes in routine settings and other developing countries. A meta-analysis of developing country ART cohorts indicated that our survival rates at 12 months may be higher than in other settings. In Khayelitsha, 13% of the cohort had died by 12 months but no patients were lost to follow-up. Across developing country cohorts, 6% had died by 12 months and 12% had been lost to follow-up giving a combined 18% dead and lost to follow-up which is 37% higher than in Khayelitsha [1]. Given that the high patient retention found in Khayelitsha would not necessarily be generalizable to other developing countries, we have conservatively increased all probabilities of dying by 37% to estimate a worst-case scenario. Our No-ART outcomes have been based on 50% survival at 24 months for patients with CD4 < 200 cells/μl whereas a review of developing country data reported median survival of 11 months across studies [37]. Death transition probabilities were adjusted accordingly in the sensitivity analysis. While inpatient utilisation was similar to published data, outpatient utilisation was higher [38]. ART visits were adjusted to be in line with recommendations in national treatment guidelines (6 during the first three months and 3 per patient-quarter thereafter) [21] and No-ART visits were decreased to 1.4 per quarter in CD4 50–199 cells/μl and 1.9 per quarter in CD4 < 50 cells/μl to be in line with published estimates. Finally, all inpatient care has been assumed to occur at secondary level hospitals given that tertiary level facilities are not accessible to the majority of South Africans.

In this alternative scenario, lifetime costs were reduced by over US$1000 for No-ART and by nearly US$4000 on ART. No-ART life expectancy decreased by 0.6 and ART life-expectancy decreased by 3.2. While lifetime costs and outcomes were reduced, the overall impact on ICERs was small (see table 6).

**Table 6:** Lifetime costs (US$), effectiveness and ICERs in alternative scenario

**Treatment option**	Lifetime costs	Outcomes		ICER	
		
		Life Years	QALYs	Life Years	QALYs
**Undiscounted**					
Generalized No-ART	1,813	2.3	1.6		
Generalized ART	9,474	9.7	8.1	1,035	1,184
**Discounted**					
Generalized No-ART	1,706	2.1	1.5		
Generalized ART	7,215	7.6	6.3	1,016	1,148

## Discussion

This study is one of the first to estimate the cost-effectiveness of comprehensive HIV care including ART based on *primary *cost, utilisation and HRQoL data from a large cohort in a developing country setting. This study has introduced a number of enhancements to standard Markov modelling approaches that have been used in developed country ART cost-effectiveness analyses. These include the creation of tunnel states to capture the rapidly diminishing utilisation and mortality through the first years on ART, and the provision of Markov states that reflect the strict two ARV-regimen approach to care contained in the WHO and South African national ART guidelines. The modelling has also introduced the concept of capturing the important cost-driver of inpatient care through *transition costs*, which are incurred as patients transition from a Markov state to death. Probabilistic sensitivity analysis has captured data uncertainty with 95% confidence intervals around lifetime costs, outcomes and ICERs.

Our findings that formed inputs to the Markov model, such as unit costs and HRQoL estimates, were comparable to other studies. Where variations between our findings and those of other studies were identified, these variables were adjusted in the sensitivity analysis. One variable that differed in our findings compared to other studies is patient utilisation rates. Although utilisation of clinic visits in this study may appear high compared to other data, it is worth reflecting on the model of care, where clinical visits were to a combined doctor-nurse team, and patients were triaged as to whether or not it was necessary for them to see a doctor at each visit. This differs from settings where visits are counted only as those in which a doctor is consulted.

While the public health setting, clinical eligibility criteria and clinical protocols used in Khayelitsha make results from this analysis highly relevant to other South African public care settings, it is possible that Khayelitsha patients had better access to HIV clinic services and other special treatment that enhanced patient retention. The multi-way sensitivity analysis, used to construct a scenario with much more conservative outcomes and lower utilisation, reveals that the main findings on the ICER are robust when these assumptions are simultaneously varied.

In a setting where access to ART is optimal, the before-and-after study design could result in a selection bias whereby patients with advanced disease who have not yet accessed ART do not adequately represent patients who never access ART. The delay between the launch of the service and the availability of ART together with the huge unmet demand for ART in this study ensured however that the pre-ART period was representative of patients who did not access ART at all. The exception is costs associated with death due to the survivor bias inherent in the design, which were explicitly addressed by the inclusion of transition costs associated with death

This study has some limitations that can be addressed in future research. Guidelines recommend that costing be undertaken from a societal perspective in order to include direct non-healthcare costs such as transport and time costs incurred by patients travelling and receiving treatment in a primary health care facility or in hospital [9]. Data from two local studies [39,47] indicate that patients incurred US$0.40 in direct travel expenses and US$0.85 in travelling time costs per visit. Across a patient's lifetime, the net present value of these costs would be US$38 and US$141 for No-ART and ART respectively. Thus while public sector HIV clinic services and tuberculosis treatment are free at the point of use in South Africa, patient costs might still pose barriers that could limit universal access to care and could have negative implication for patient retention and adherence. Government policy [48] has indicated a willingness to provide patient transport or to subsidize patient costs in certain circumstances but this policy has yet to be implemented. We therefore recommend that additional research be undertaken into the full range of non-healthcare costs imposed on patients in order to inform policy development in this area.

The further interpretation of cost-effectiveness results in terms of allocative efficiency requires caution. Generally, this would require an assessment of whether QALYs gained on ART were a good buy given the level of the ICER, either by reference to government policy on the maximum willingness to pay for a QALY or by comparing to other published cost per QALY analyses. In South Africa, lack of government policy precludes the first option and paucity of local cost per QALY studies precludes the second. However, the Commission on Macroeconomics and Health [49] has recommended that an ICER below per capita gross domestic product (GDP) could be considered to be very cost-effective. South Africa's GDP of US$ 3,089 [50] puts ART well below this threshold with the implication that ART is allocatively efficient. In addition, if ARV price reductions are achieved in future government ARV tenders, the cost-effectiveness of ART will be further enhanced.

We would argue however that equity is as important as efficiency when allocating resources to HIV treatment. Over six million South Africans are HIV-positive and will be in need of treatment within the next 10 years with the implication that an equity goal of universal access for all in need by 2010 is a formidable challenge. We therefore recommend additional research into the cost-effectiveness of less resource intensive ART options such as nurse driven as opposed to doctor driven treatment, possibly limiting access to first-line [51] and/or limiting or simplifying expensive laboratory testing. While these options might be less effective, in instances where there are financial constraints to universal access to ART, social values should determine whether we as Africans think it is fairer to offer comprehensive treatment to fewer patients or universal access to a more limited package of benefits. The very question of financial constraints becomes a political and societal value question in the context of the extent of morbidity and mortality being faced in Southern Africa as a consequence of HIV.

## Conclusion

Universal ARV provision has major resource implications for developing countries, particularly in Africa. To date, decisions on ARV programs have been made in a context of extremely limited economic primary-data evidence. This paper provides detailed results (from analysis of primary data) on utilisation of healthcare on and off ART, full economic costs of HIV-related services, primary outcomes on ART over 48 months and extrapolated lifetime costs, LYs and QALYs, which could contribute to evidence-informed decision-making. While the ICER for ART compared to No-ART in this research provides strong support for the South African government's universal ARV treatment policy, in the context of our level of economic development, aspects of the treatment guidelines (e.g. less resource intensive models of care and regimens) should be further explored if ART is to be equitably scaled-up and universal access achieved.

## Competing interests

The author(s) declare that they have no competing interests.

## Authors' contributions

SC and AB collected and analysed primary data, and designed and analysed the Markov models. DM contributed to the development of the methods. SC wrote the first draft of the paper. All authors read and approved the final manuscript.
